# Programmed cell death in atherosclerosis and vascular calcification

**DOI:** 10.1038/s41419-022-04923-5

**Published:** 2022-05-18

**Authors:** Min Li, Zhen-Wei Wang, Li-Juan Fang, Shou-Quan Cheng, Xin Wang, Nai-Feng Liu

**Affiliations:** Department of Cardiology, Zhongda Hospital, School of Medicine, Southeast University, Nanjing, 210009 PR China

**Keywords:** Calcification, Cell death

## Abstract

The concept of cell death has been expanded beyond apoptosis and necrosis to additional forms, including necroptosis, pyroptosis, autophagy, and ferroptosis. These cell death modalities play a critical role in all aspects of life, which are noteworthy for their diverse roles in diseases. Atherosclerosis (AS) and vascular calcification (VC) are major causes for the high morbidity and mortality of cardiovascular disease. Despite considerable advances in understanding the signaling pathways associated with AS and VC, the exact molecular basis remains obscure. In the article, we review the molecular mechanisms that mediate cell death and its implications for AS and VC. A better understanding of the mechanisms underlying cell death in AS and VC may drive the development of promising therapeutic strategies.

## Introduction

AS and VC are closely related to decreased vascular compliance, which can cause plaque rupture and thrombosis. VC is defined as abnormal deposits of mineral matrix in the vessel wall [1, 2]. It is a common pathological feature of vascular injury such as AS, diabetes, and chronic kidney diseases. Calcification can occur in the intimal and medial layers of the vessel wall, and valvula [3]. Intimal calcification is mainly involved in the calcification of atherosclerotic lesions. Medial calcification is primarily driven by vascular smooth muscle cells (VSMCs), which is similar to bone formation. In the pathological microenvironment, VSMCs can lose contractility markers and undergo the phenotypic transition to chondrocytic, osteoblastic, and osteocytic cells [4]. Initially, VC was regarded to be the result of passive degenerative processes, and now it is illustrated that VC is a complex and actively regulated process mediated by a range of molecular signaling pathways [5, 6]. Thereinto, several signaling pathways involved in specific forms of cell death play a significant role in VC.

Cell death, an inevitable destiny of all life, is crucial to organismal homeostasis. When it is dysregulated, this will lead to a series of pathological consequences. Regulated cell death relies on specialized molecular machinery and differs from classic necrosis that is unregulated cell death caused by overwhelming physical, chemical, or biological insults [7]. Programmed cell death (PCD) as a subset of regulated cell death includes classical apoptosis in the context of development and tissue homeostasis, and other forms that occur in the microenvironment of exogenous or endogenous perturbations, such as necroptosis, pyroptosis, autophagy, ferroptosis, etc [8, 9]. A growing body of evidence suggests that different PCD pathways are interrelated at multiple levels and have special effects on health and disease. Therefore, we summarize the molecular and functional relationships between different PCD pathways, emphasizing that these pathways are complex and extended system, which responds to a series of challenges from the body, and plays an important role in the occurrence and development of AS and VC.

## Apoptosis

### Apoptotic signaling

**Fig. 1:**
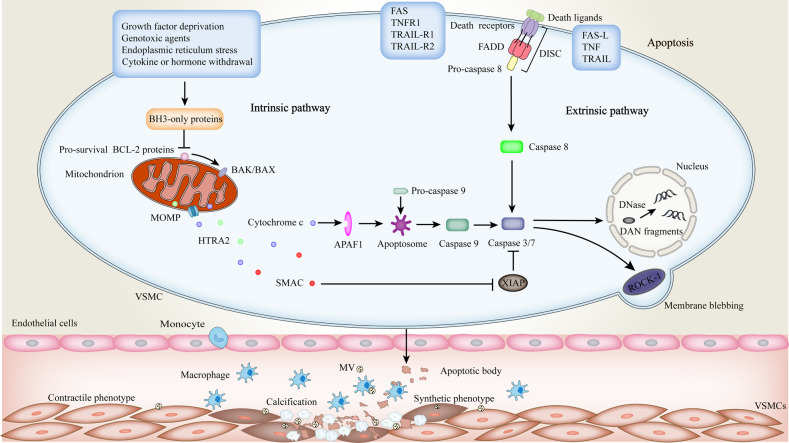
Apoptotic signaling in VC. Apoptosis can be triggered by intrinsic and extrinsic pathways. BH3-only proteins have crucial roles in intrinsic apoptosis, which can bind to anti-apoptotic proteins and inhibit their function, thereby inducing mitochondrial outer membrane permeabilization (MOMP). Afterward, released cytochrome *c* binds apoptotic peptidase activating factor 1 (APAF1) and leads to the formation of apoptosome. The caspase 9 is activated in this complex, in turn, it activates the executioner caspase 3/7. The extrinsic apoptotic pathway is initiated by ligand‐receptor binding, and then these death receptors can recruit caspase-binding adapter proteins to form the “death-inducing signaling complex (DISC)”, which activates caspase 8 and downstream effector caspases 3 and 7. The executioner caspases further cause internucleosomal DNA fragmentation, membrane blebbing, and apoptotic body formation. Macrophages can recognize the ‘eat me’ signals on the surface of these bodies, such as phosphatidylserine (PS), to mediate efferocytosis. Whereas, lipid metabolism disorder and calcium-phosphate imbalance can give rise to phagocytic dysfunction of macrophages and increased release of pro-calcific matrix vesicles. Furthermore, apoptotic bodies serve as nucleation points for calcium crystals with the deposition of hydroxyapatite. Together, these changes lead to vascular mineralization.

Apoptosis is the most deeply studied form of PCD. Cell death under physiological conditions is mainly carried out by apoptosis which is a non-inflammatory or silent process. Apoptosis is characterized by a series of morphological changes. Apoptotic cells can be further coordinated to disintegrate into smaller fragments, the so-called apoptotic bodies (ABs), which ensure the sequestration of intracellular components [10]. Subsequently, those can be quickly phagocytosed by macrophages to prevent the release of inflammatory factors. Two pathways of apoptosis include the intrinsic pathway involving mitochondria, also referred to as the B cell lymphoma-2 (BCL-2) regulated pathway, and the extrinsic pathway initiated by death receptors (Fig. 1).

The mitochondrial pathway of apoptosis is triggered by various stimuli, such as growth factor deprivation, genotoxic agents, endoplasmic reticulum stress, and cytokine or hormone withdrawal, which act on inducing transcriptional and post-translational increases in the expression of pro-apoptotic members of the BCl-2 family, called BH3-only proteins (for example, BID, BMF, PUMA, NOXA, BIM, and BAD) (Fig. 1) [11]. They can directly or indirectly antagonize the pro-survival BCL-2 proteins (such as BCL-2, BFL-1), thereby unleashing BAK and BAX, crucial effectors of apoptosis, that mediate mitochondrial outer membrane permeabilization (MOMP) and cause the release of apoptogenic factors including cytochrome *c*, second mitochondrial activator of caspases (SMAC)/DIABLO and HTRA serine peptidase 2(HTRA2)/OMI [12, 13]. Thereinto, cytochrome *c* can bind to apoptotic peptidase activating factor 1 (APAF1), leading to a change in its conformation, followed by the exposure of caspase recruitment domains (CARD) and oligomerization domains that allow several APAF1s to assemble into a complex termed the apoptosome [14]. In this complex, the procaspase 9 is recruited and activated, which then promotes the activation of the downstream executioner caspases 3 and 7 [15]. The X-linked inhibitor of apoptosis (XIAP), one of the inhibitors of apoptosis proteins, can attenuate the activation of the above-mentioned caspase cascade [16].

The other pathway triggered by death receptors intersects with the mitochondrial pathway to collectively mediate apoptosis. The death receptor pathway of apoptosis is activated by the binding of specific ligands to their homologous cell surface receptors (Fig. 1). This results in the recruitment of adaptor proteins such as FAS-associated death domains (FADD), or TNF receptor 1(TNFR1)-associated death domains (TRADD), through the death domains (DD) present in both the receptors and their adaptors [17]. Furthermore, the homologous interactions between death effector domains of FADD and procaspase 8 facilitate the recruitment of procaspase-8 into the death-inducing signal complex (DISC), which causes the activation of the initiator caspase 8 then cleaving and activating downstream effector caspases 3 and 7 [18]. The effector caspases activated by either pathway can induce the characteristic apoptotic morphology, including membrane blebbing caused by the actin contraction due to the direct or indirect activation of ROCK-1 kinase by those caspases, and DNA cleavage and nuclear condensation derived from caspase-activated DNase [19]. Subsequently, as the apoptotic cells dissociate into ABs, macrophages recognize the ‘eat me’ signals on the surface of these bodies, such as phosphatidylserine (PS), to mediate efferocytosis [20].

### Apoptosis in AS

The level of apoptosis is closely related to the stage of atherosclerotic lesions and plaque rupture. Few apoptosis was found in the stage of adaptive intimal thickening and fat stripes, while apoptotic foci were present in advanced lesions [21]. In fact, under physiological conditions, the clearance of apoptotic cells occurs when the immune system is not activated, and is mediated by various phagocytes [22]. The imbalance of apoptotic cell clearance can lead to the development of secondary necrosis of apoptotic cells, followed by the release of intracellular contents, promoting the occurrence of chronic inflammatory diseases such as AS [23]. During disease progression, the consequences of apoptosis depend primarily on the cell type involved, the location of the apoptotic cells, and the stage of the plaque [24]. Endothelial cells (ECs) covering the entire vascular network form a barrier that controls the communication of biomolecules and immune cells between the circulation and tissues, which are believed to be crucial participants in the initiation and progression of atherosclerosis [25]. Multiple factors in AS have been shown to induce apoptosis, including oxidized-LDL (ox-LDL), oxidative stress, hyperglycemia, and cholesterol overload, etc. [21]. Among them, ox-LDL induces ECs apoptosis in a Fas ligand-dependent manner [26]. Nitric oxide is synthesized and secreted by ECs, which can counteract apoptosis by inhibiting IL-1α-converting enzyme-like and cysteine protease protein-32-like proteases [27]. However, reactive oxygen species (ROS) can cause endothelial dysfunction and apoptosis through the inactivation of nitric oxide [28]. High glucose induces NF-κB-dependent upregulation of cyclooxygenase-2 through the PI3K/Akt signaling pathway, which in turn triggers caspase-3 activity that promotes apoptosis of human ECs [29]. Finally, the death of ECs can initiate plaque erosion. Additionally, apoptosis of ECs can contribute to an increase in procoagulant activity through redistribution of PS to the cell surface and loss of anticoagulant surface components, causing the occurrence of thrombotic events [30].

Next, apoptosis of macrophages is involved in different stages of atherosclerotic plaque. In early lesions, apoptosis of macrophages would limit plaque development by negatively regulating inflammation. As the disease progresses, in advanced plaques, defective efferocytosis and the ensuing accumulation of apoptotic cells hinder inflammatory resolution, thus accelerating plaque necrosis or rupture, which is a key factor in acute luminal thrombosis [31]. Evidence to support this theory comes from the study by Gautier et al., suggesting that increased susceptibility of macrophages to apoptosis can result in a decrease in plaque size after 5 weeks of the Western diet, but an increase in plaque size after 15 weeks of the Western diet [32]. Normally, the observation window of apoptosis is narrowed due to the timely clearance mechanism. However, in advanced atherosclerotic lesions, increased cell death stems from ubiquitous apoptosis trigger-oxLDL, which competes with apoptotic cells for clearance, thereby impairing exocytosis [24]. Furthermore, the transformation of macrophages into foam cells also weakens the capacity to engulf apoptotic debris [33]. Ultimately, the combined effect of many factors causes the dichotomous model of macrophage death.

VSMC apoptosis is a key process that determines plaque stability. VSMCs in plaques mainly originate from the media layer of the vascular wall and are the main source of collagen production in the fibrous cap, which is beneficial to plaque stability and responsible for its tensile strength [34]. Apoptosis of VSMCs can lead to thinning of the fibrous cap and promote the formation of necrotic core, plaque rupture, and calcification [35]. Apoptotic levels of VSMCs are low in early plaques but increase with the progression of lesions, which is why VSMC death can distinguish the initial fatty streaks from fibroatheroma. Fatty streaks are cell-rich and mainly composed of VSMCs, whereas the lipid core of fibroatheroma is predominantly acellular due to the gradual loss of VSMCs in situ [34]. Subsequently, the migrated VSMCs proliferate and restore the lipid core to form a fibrous cap. However, VSMC apoptosis induced by a variety of pathogenic factors can cause the fibrous cap to become smaller and thinner, resulting in the formation of unstable plaques [36]. VSMC apoptosis is always located in areas with high levels of inflammation, and it is speculated that inflammatory cells or pro-inflammatory cytokines may sensitize VSMCs to apoptosis [37]. In fact, unlike many cell types, the death receptor Fas/TNFR1 expressed by VSMCs is sequestered in the Golgi apparatus, making cells relatively resistant to Fas-induced apoptosis [38]. After specific stimulation, such as pro-inflammatory cytokines (interleukin-1β, tumor necrosis factor α (TNF-α), and interferon-γ (IFN-γ)), oxLDL, nitric oxide, and free radicals, etc., Fas can be relocated to the cell surface to initiate VSMC death [36, 39, 40]. VSMC apoptosis in advanced plaques can also promote plaque thrombogenicity by exposing PS on the surface of apoptotic cells [41]. In addition, the remnants of apoptotic VSMCs are present in plaques in the form of matrix vesicles (MVs) and can serve as nucleation structures for bone formation, leading to plaque microcalcification [35]. Moreover, inefficient clearance of apoptotic VSMCs can trigger secondary necrosis and amplify plaque inflammation, creating a vicious cycle [34].

### Apoptosis in VC

Intimal calcification is a common early event in the pathogenesis of atherosclerosis. Calcification can also occur in the media of vessel wall, which is an effective predictor of future cardiovascular events in asymptomatic patients [42]. During the bone formation and cartilage mineralization, MVs are thought to initiate calcification [35]. Similarly, MVs have been found in calcified arteries and heart valves [43, 44]. Subsequently, Kockx et al. demonstrated that in advanced atherosclerotic plaques, these vesicle-like structures were derived from VSMCs and contained BAX proteins, suggesting that they were apoptotic remnants [45]. Apoptosis plays an indispensable role in the initiation of VC. Firstly, the study revealed that stimulation of apoptosis in human VSMCs with a combination of anti-Fas IgM and cycloheximide increased nodule calcification, whereas inhibition of apoptosis with the cell-permeable caspase-inhibitor ZVAD.fmk decreased calcification [35]. Secondly, the DNA breaks characteristic of apoptosis was found on day 7 of VSMC nodule culture, but the deposition of calcium crystals could not be detected until day 28 [35]. This implies that apoptosis precedes calcification and the calcification process in nodules is regulated. Finally, it is revealed that ABs and MVs are involved. It turns out that VSMC-derived ABs accumulate calcium through an integral AB membrane. When ABs expose PS, potential calcium-binding sites and membrane surfaces suitable for hydroxyapatite deposition are formed; Nevertheless, no calcium accumulation was observed when the AB membrane was permeabilized with NP-40 [35]. Actually, in the early stage, phagocytes within the nodules may recognize and phagocytose Abs, but in the later stage, older nodules contain less efficient phagocytes, allowing the ABs to stimulate the growth of calcium crystals [46]. Besides this, MVs produced by VSMCs are also involved in the regulation of VC [47]. VSMCs have been proved to be a heterogeneous group of cells with multiple differentiation potentials. VSMCs with contractile phenotype can actively secrete MVs to regulate the microenvironment and prevent VC, while synthetic phenotype accelerates calcification [48, 49]. MVs secreted by healthy VSMCs contain endogenous calcification inhibitors, such as circulating fetuin-A and vitamin K-dependent matrix GLA protein (MGP), to counteract calcification [48]. Importantly, calcification also is significantly related to mineral imbalances [50]. Fetuin-A loaded into MVs can bind and stabilize minerals, preventing lesion expansion [51]. Subsequently, macrophages are involved in the uptake of fetuin A-containing particles through the surface scavenger receptor-AI/II [52]. However, long-term exposure of MVs to the metastable supersaturated solution of calcium and phosphate facilitates calcium influx and the formation of Annexin (Anx)-PS complexes as the nucleation cores, and even further mineralizes the components of extracellular matrix (ECM) (Fig. 1) [53, 54]. Large calcification is caused by the accumulation of microcalcifications. The microcalcification under the vulnerable atherosclerotic plaque accelerates the rupture of plaque, while the large calcification under the stable fibrous cap maintains the stability of the plaque [55]. In patients with chronic kidney disease (CKD), the severe storm of metabolic dysfunction results in fewer calcification inhibitors and a higher proportion of calcification-related markers such as Gla-rich proteins (CRP) in MVs, which accelerates the calcification of the intima and media [56]. The production of MVs and the recycling of fetuin A are regulated by the sphingomyelin phosphodiesterase 3 (SMPD3) that is a key mediator of biomineralization [48]. The inhibition of this factor reduces the release of MVs and calcification, which perhaps have certain therapeutic potential.

## Necroptosis and pyroptosis

### Necroptotic and pyroptotic signaling

**Fig. 2:**
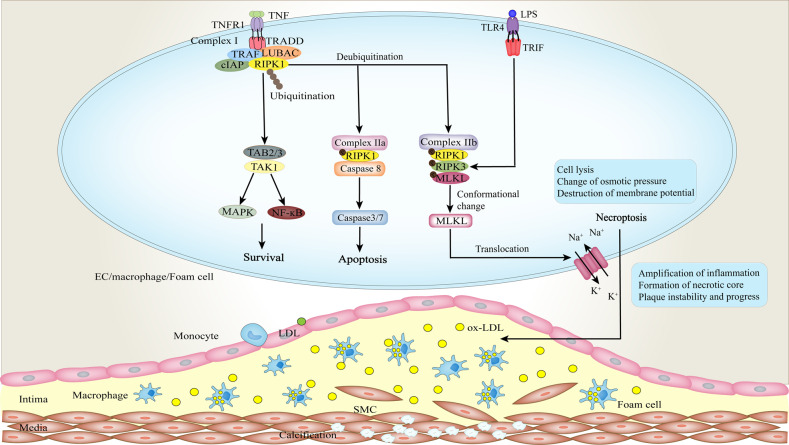
Necroptotic signaling in AS. Necroptosis is commonly induced by the binding of tumor necrosis factor (TNF) to its TNF receptor 1 (TNFR1). Afterward, TNFR1 recruits the adaptor protein TRADD which functions as a platform for recruiting other proteins and forming complex I. The deubiquitination of receptor-interacting protein kinases 1(RIPK1) results in the formation of complex IIb, activating the necroptotic signaling cascade. Here, the autophosphorylation of RIPK1 facilitates the phosphorylation and activation of RIPK3, which in turn mediates the phosphorylation and conformational changes of mixed-line kinase domain like (MLKL), eventually causing plasma membrane rupture. In early atherosclerotic diseases, high levels of RIPK1 drive NF-κB-dependent inflammation and the activation of ECs. Activated ECs secrete more adhesion molecules to attract and recruit monocytes and other inflammatory cells. Next, ox-LDL induces RIPK3 expression in macrophages and foam cells, promoting cellular necroptosis and the forming of necrotic lipid pool. Ultimately, this leads to plaque instability and rupture, as well as a variety of cardiovascular events.

Necroptosis and pyroptosis differ from necrosis, as the lytic and inflammatory types of programmed cell death, require the mixed-line kinase domain like (MLKL) and the membrane-damaged gasdermin D (GSDMD) proteins, respectively, to mediate the release of pathogen-associated molecular patterns (PAMPs), endogenous damage-related molecular patterns (DAMPs) and pro-inflammatory molecules. Death receptor signaling does not specifically trigger apoptosis; it also promotes cell survival or necroptosis (Fig. 2) [7, 57]. Apoptosis depends on the activation of caspase, whereas necroptosis requires continuous activation of receptor-interacting protein kinases 1(RIPK1) to 3 (RIPK3), then triggering the key executor of necroptosis—MLKL [58]. Necroptosis is commonly induced by the binding of tumor necrosis factor (TNF) to its TNF receptor 1 (TNFR1), but it can also mediate by other factors, such as tumor necrosis factor-related apoptosis-inducing ligand (TRAIL) targeting its TRAIL receptor, and Fas ligand (Fas-L) for its Fas receptor [17, 59]. Subsequently, TRADD is recruited to the tail of TNFR1 through interactions between their DD, which further promotes the recruitment of RIPK1 and directs the formation of the protein complexes at the intracellular plasma membrane [60]. The DISC has been mentioned above. Complex I is composed of a variety of proteins, such as RIPK1, adaptor proteins TNF receptor-associated factors 2 and 5 (TRAF2 and TRAF5), linear ubiquitin chain assembly complex (LUBAC), a cellular inhibitor of apoptosis proteins 1 and 2 (cIAP1 and 2), and deubiquitinases, etc[61]. RIPK1 is polyubiquitinated in complex I, and its deubiquitination leads to the formation of either complex IIa or IIb. In the pro-survival signaling, nuclear factor κ-light-chain-enhancer of activated B cells (NF-ĸB) targets transcriptionally cIAP1 and 2, XIAP, cellular-FADD like interleukin-1-converting enzyme-inhibitory protein(cFLIP), and BCL-2 to play a key role [62–64]. In contrast, the type of complex II (a or b) and the state of caspase 8 (activated or inactivated) determine the transition to apoptosis or necroptosis [65]. Apoptosis occurs in a RIPK1-independent or RIPK1-dependent manner, which is mainly affected by the ubiquitination and phosphorylation states of RIPK1 [64, 66]. Activated caspase-8 can mediate the cleavage of RIPK1 and RIPK3, and prevent necroptosis.

Necroptosis initiates when the activity of caspase 8 is blocked or apoptosis is inhibited. The kinase activity of RIPK3 is necessary for necroptosis, which is activated by phosphorylation of RIPK1, or Toll-like receptor (TLR) recruiting the adaptor protein—Toll-interleukin-1receptor (TIR) domain-containing adapter-inducing interferon (TRIF) [67, 68]. RIPK3-induced phosphorylation of MLKL causes its conformational changes to expose the helical bundle, which results in its translocation and plasma membrane permeability (Fig. 2) [69, 70]. In fact, even in the absence of RIPK3 activation, the forced polymerization of MLKL can also lead to permeabilization of the plasma membrane [71, 72]. The underlying mechanism of plasma membrane permeability perhaps involves the binding of MLKL to phosphatidylinositol phosphate, the changes in osmotic pressure, and the destruction of membrane potential [19, 70, 73]. In addition, activated MLKL promotes the formation of disulfide bond-dependent amyloid fibers, and however, whether this contributes to the recruitment of additional necroptotic factors to the cellular membrane or directly participates in membrane binding and damage remains murky [72].

**Fig. 3:**
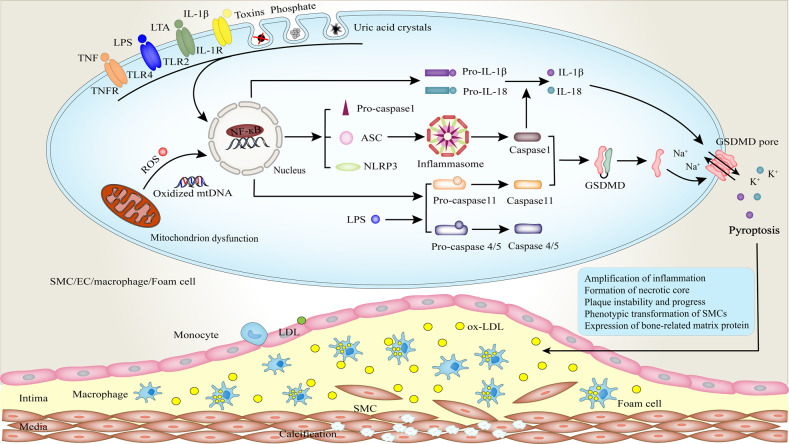
Pyroptotic signaling in AS and VC. Cell recognizes a variety of stressors, such as DAMPs, PAMPs, uric acid crystals, toxins, phosphate, reactive oxygen species (ROS), etc., to undergo inflammasome formation and pyroptosis. Here, inflammasome formation requires at least two steps including priming and activation. Canonical NLRP3 gene transcription is triggered by the pattern recognition receptors (for example, TNFR, TLR, IL-1R) located at the cell surface. Then, the inhibitor of NF-κB (IκB) is phosphorylated and activated, and the functional NF-κB is released into the nucleus. As a result, the expression of pro-IL, pro-caspase11, and the inflammasome components (NLRP3, pro-caspase1, and ASC) is upregulated. The inflammasome is assembled. However, its activation mechanism remains largely unknown. Next, activated caspase-1 cleaves the cytokine precursors pro-IL-1β and pro-IL-18 into mature proinflammatory factors IL-1 and IL-18. In addition, it also mediates the cleavage of GSDMD to form the N-terminal pore-forming domain, which translocates to the cellular membrane, where it forms membrane pores and drives the release of pro-inflammatory cytokines. Persistent stimulation of the inflammatory leads to ECs damage, increased ROS generation, enhanced adhesion and migration of monocytes, foam cell necrosis, and VSMC proliferation and osteogenic differentiation, which facilitates AS and VC.

Cells can also recognize a variety of stressors, such as DAMPs, PAMPs, uric acid crystals, toxins, phosphate, ROS, etc., to undergo inflammasome formation and subsequent pyroptosis (Fig. 3) [74, 75]. PAMPs usually contain lipopolysaccharide (LPS), viral double-stranded RNA (dsRNA), peptidoglycan (PGN), lipoteichoic acid (LTA), and others, while DAMP includes misfolded or aggregated proteins, metabolites [76, 77]. The assembly of inflammasomes relies on NOD-like receptor (NLR) family pyrin domain-containing 3 (NLRP3) to recruit the apoptosis-associated speck-like protein containing a CARD (ASC), which in turn recruits and activates the caspase1 [7]. Its expression requires at least two steps including priming and activation. Priming involves activated receptors including TLR2/3/4, interleukin-1 receptor (IL-1R), or TNFR1 to up-regulate the expression of NF-κB dependent target genes (pro-IL-1β, pro-IL-18, NLRP3, ASC, procaspase1, and procaspase11) through the corresponding adaptor proteins, such as myeloid differentiation primary response 88 (MyD88), IL-1R-associated kinase 1 (IRAK-1), or TRIF [78, 79]. However, the specific mechanism of inflammasome activation is largely unknown. Currently, the following reasons are mainly considered: 1) potassium efflux induced by that MLKL form pores [76]; 2) Cl^−^ outflow through the chloride intracellular channel (CLIC) [80]; 3) lysosomal rupture [81]; 4) mitochondrial or metabolic dysfunction [82, 83]; 5) the combination of phospholipid phosphatidylinositol-4-phosphate (PtdIns4P) and NLRP3 [84]. Next, the activated caspase-1 reprocesses pro-IL-1β and pro-IL-18 into mature IL-1 and IL-18. More interestingly, necroptotic signaling can trigger a RIPK3-MLKL-NLRP3-Caspase-1 axis, leading to the maturation of IL-1β [85, 86]. Mechanically, data support that potassium efflux caused by the membrane-associated MLKL triggers the NLRP3 signaling [85]. Furthermore, LPS can directly bind to and activate the caspase 11 and its human orthologues caspases 4 and 5 in a noncanonical way [87, 88]. Although caspase 11 cannot convert pro-IL-1β and pro-IL-18 into their active forms, its proteolysis of GSDMD is similar to that of caspase1.

### Necroptosis and pyroptosis in AS

Continuous stimulation of inflammatory response is harmful to the vessel wall. The presence of inflammatory factors plays a crucial role in all phases of AS [89], which promotes the occurrence of VC. In early atherosclerotic diseases, ECs express high levels of RIPK1 to drive NF-κB-dependent inflammation [90]. ECs exposed to the blood circulation are the first cells of vessel wall to sense endogenous danger signals (such as hyperlipidemia, hyperglycemia, hypertension, inflammatory mediators, ROS, and others), whose activation is the first and most important step [91]. In the dyslipidemic and inflammatory environment, the caspase 1-inflammasome pathway in ECs can perceive the increased lipids and inflammatory factors to trigger pyroptosis and activate ECs (Fig. 3) [92]. Activated ECs secrete adhesion molecules to attract and recruit monocytes and other inflammatory cells. Diabetes also is a risk factor related to vascular dysfunction [93]. The high glucose state in diabetes can strongly deteriorate endothelial damage and necroptosis. Hyperglycemia significantly enhances the expression of RIPK3 in ECs [94]. Dying ECs cause a decrease in their number and integrity and increase the permeability of endothelial monolayer, which promote the deposition of lipids, and the migration of monocytes and VSMCs to the intima, further damaging the vasculature (Figs. 2, 3) [95].

The deposition and oxidation of low-density lipoproteins (LDL) are considered to be the initiating agent of AS [96]. Ox-LDL deposited in the endothelium, on the one hand, can induce the expression of RIPK3 in macrophages, facilitate the phosphorylation of RIPK3 and MLKL, and cause the necroptosis of macrophages (Fig. 2) [97]; On the other hand, it also triggers the formation of NLRP3 inflammasome and resultant production of caspase 1, thereby mediating the pyroptotic cell death of macrophages (Fig. 3) [98]. The increase in macrophage death causes severe inflammation and lesion instability. Moreover, macrophages phagocytose and internalize cholesterol crystals, transforming into lipid-rich foam cells, which is the hallmark of arterial fatty streaks. In the process, when macrophages are unable to digest those crystals and die, more destructive substances will be released, which recruits monocytes and VSMCs, and amplifies vascular inflammation. This is a vicious circle. Subsequently, pro-inflammatory cytokines repeatedly trigger foam cells to undergo the process of necroptosis and pyroptosis, forming a lipid-laden pool within the lesion (Figs. 2, 3) [99, 100]. Studies confirm that the death of these cells is closely related to the formation of necrotic cores and plaque instability in advanced atherosclerotic lesions [97, 100]. The migration of VSMCs from the media into the intima is a pivotal feature of arteriosclerosis. TNF-α can induce inflammasome priming in human VSMCs by upregulating the expression of NLRP3 [101]. Caspase1-dependent pyroptosis may be involved in the death of VSMCs. Pyroptosis in VSMCs results in the loss of collagen and matrix, which further weakens the fibrous cap and promotes plaque instability [95]. However, the evidence regarding VSMC necroptosis in AS and VC is insufficient, and the exact relationship between pyroptosis of VSMCs and AS needs also to be further established.

### Pyroptosis in VC

VC resembles bone development, which is a pathological process. Medial calcification occurs in close relation with VSMCs that are the primary cell type constituting the arterial wall. Prior researches have established that VSMCs express bone formation-related transcription factors including Msx2, Runx2, and Osterix under the action of calcification stimulators such as inflammatory factors, high phosphorus, and high glucose [102]. Next, newly acquired evidence indicates that NLRP3 inflammasome plays an integral role in the development of VC. VC is a chronic inflammation event. The levels of IL-1β are significantly elevated in both calcified VSMCs and aortas [103]. Targeting the classical NF-κB/NLRP3 inflammatory pathway can inhibit calcification through plasma trimethylamine N-oxide (TMAO) and puerarin [103, 104]. In addition to this, inhibition PI3K-AKT/ERK/NF-κB/NLRP3 signaling pathway also blocks the calcification of aortic valve interstitial cells [105]. This elucidates that activated NLRP3 inflammasome is required for calcification. Phosphate stimulation can up-regulate the activity of inflammasome, and the resultant expression of caspase1and IL-1β, which drives VSMC calcification [75]. Activation of NLRP3 inflammasome induced by calcium phosphate crystals occurs in the early stage of cultured cells, implicating that the signaling may be an acute response to small calcium phosphate crystals or the mineral deposits during calcification. Inhibition of NLRP3 with short hairpin RNA can prevent VSMC calcification, highlighting the potential importance of NALP3 signaling in regulating VC.

More importantly, VC is the leading cause of high mortality from cardiovascular complications in diabetes. High concentrations of glucose can increase IL-1β levels by upregulating the expression of the thioredoxin-interacting protein, one of the NLRP3-binding proteins [106]. High glucose-mediated VC is mainly related to the activation of Akt/ROS/NLRP3/caspase 1/IL-1β axis that induces the expression of bone-related matrix protein, such as alkaline phosphatase (ALP), osteopontin (OPN), and osteocalcin (OCN) in VSMCs [107]. Inhibition of NLRP3 inflammasome activation and subsequent reduction of IL-1β secretion can ameliorate insulin resistance and VC [106, 107]. These data underscore the importance and regulation of NLRP3 inflammasome-dependent system in hyperglycemia and VC.

## Autophagy

### Autophagic signaling

**Fig. 4:**
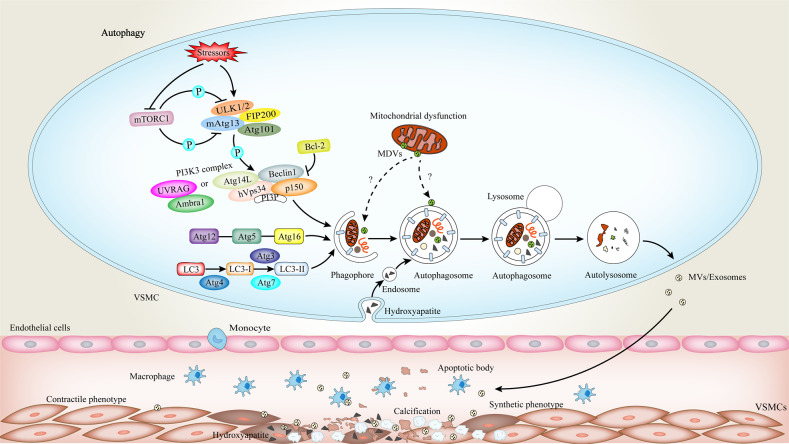
Autophagic signaling in VC. Autophagy is activated by multiple stressors. Subsequently, the uncoordinated-51-like protein kinase 1/2 (ULK1/2) complex dissociates from the mammalian target of rapamycin complex 1 (mTORC1) and becomes activated to initiate the formation of a phagophore. Phosphatidylinositol-3-phosphate (PI3P) promotes autophagosomal membrane nucleation. After, the elongation of the phagophore involves two ubiquitin-like conjugation systems: the Atg5-Atg12-Atg16L and the microtubule-associated protein 1 light chain 3-phosphatidylethanolamine (LC3-PE). This leads to the formation of autophagosomes. Finally, autophagosomes fuse with lysosomes to form autolysosomes where cargoes are degraded by lysosomal enzymes. Autophagy deficiency or lysosomal dysfunction promotes VC. The calcification precursors like calcium and phosphate are formed or processed in the endosome, autophagosomes, or autolysosomes, which participates in the regulation of intracellular calcium and phosphate homeostasis. Hydroxyapatites enter the cell by endocytosis, via endosomes. Then these endosomes fuse with autophagosomes and are transported to autolysosomes where they can be degraded or released into the ECM by packaging in the MVs. Impairment of lysosome function results in increased secretion of pro-calcific extracellular vesicles, promoting VC.

Autophagy is an evolutionarily conserved process that is essential for maintaining cell homeostasis. At present, three main types of autophagic response have been confirmed: macroautophagy, microautophagy, and chaperone-mediated autophagy, of which macroautophagy is mainly responsible for the targeting of organelles. The core of this process is the formation of double-membrane vesicles called autophagosomes, which are responsible for transferring longevity proteins and excess or damaged organelles into lysosomes to degrade, and reuse the resulting macromolecules [108]. Here, we focus on macroautophagy, which is henceforth termed autophagy. In response to stressors including nutrient starvation, oxidative stress, hypoxia, increased phosphate levels, and others, autophagy levels can be significantly increased as a cytoprotective mechanism, leading to adaptation and survival [109]. The autophagic molecular machinery was first identified in yeast followed by recognition of homologs in humans. To date, at least 40 autophagy-related genes (Atg) have been discovered. Environmental stresses inactivate the mammalian target of rapamycin complex 1 (mTORC1) to induce the activation of uncoordinated-51-like protein kinase 1/2 (ULK1/2) complex that interacts with mAtg13, FIP200 (an ortholog of yeast Atg17), Atg101, resulting in phagophore assembly (Fig. 4) [110]. Afterward, phosphatidylinositol-3-phosphate (PI3P) promotes autophagosomal membrane nucleation. The substance is produced by a class III phosphatidylinositol 3-kinase (PI3K3) complex which consists of Atg14L (a homolog of Atg14), Beclin1 (a homolog of Atg6), hVps34 (a homolog of vacuolar protein sorting 34(Vps34)), and p150 (a homolog of Vps15), and its activity is regulated by Bcl-2 [108]. UVRAG (a homolog of Vps38), another member of this type of complex, can compete with Atg14L for Beclin 1 binding to participate in autophagy regulation [111]. Moreover, it interacts with Bif-1 (Bax interaction factor 1), which further induces membrane deformation [112]. Autophagy/Beclin 1 regulator 1 (Ambra1) is also essential for the induction of autophagy through direct interaction with UVRAG [113]. Subsequently, phagophore elongation requires two ubiquitin-like conjugation systems—Atg5-Atg12-Atg16L and microtubule-associated protein 1 light chain 3(LC3) [114]. Under the synergistic action of Atg4, Atg7, and Atg3, the precursor of the LC3-like protein is further cleaved into the mature form that binds to phosphatidylethanolamine (PE) and is recruited into autophagosome [115]. Eventually, the autophagosome fuses with the lysosome and the contents are degraded. In selective autophagy, LC3 interacts with autophagic adaptor (such as p62, ALFY, or others) to sequester specifically labeled cargo (e.g., peroxisomes, mitochondria, ribosomes, liposomes, etc.) into autophagosome for delivery and degradation [110].

### Autophagy in AS

Autophagy can regulate endothelial homeostasis, VSMC phenotypic transition, and calcium (Ca^2+^) homeostasis, which plays a key role in maintaining cellular metabolism and normal vascular cell function [116]. In atherosclerotic disease, autophagy deficiency accelerates atherogenesis and plaque instability. Specifically, in primary ECs, native or modified lipids stimulate the formation of autophagosomes. However, the deletion of autophagy ATG7 gene or the addition of lysosomal inhibitor chloroquine can lead to intracellular accumulation of oxidized LDL and increase atherosclerotic burden [117]. This accumulation of lipids is not taken up and delivered to lysosomes through receptor-mediated endocytosis but is closely associated with the endosome and autophagosome pathways [117, 118]. Intact endothelial autophagy is necessary for maintaining vascular lipid homeostasis. In addition, AS is prone to occur at arterial bifurcations and bends, where blood flow is low or disordered, and autophagy efficiency is reduced, which accelerates EC inflammation, apoptosis, and aging, and is also conducive to the development of atherosclerotic lesions. Even in areas with high shear stress, known as atheroresistant regions, the deletion of EC-specific autophagy ATG5 gene can also result in massive atherosclerotic plaque formation [119].

Autophagy deficiency also increases apoptosis and oxidative stress in lesional macrophages, reduces the phagocytic clearance of apoptotic cells, and results in secondary necrosis and the amplification of plaque inflammation [120, 121]. Oxidative and endoplasmic reticulum stress are the main causes of apoptosis. During disease progression, macrophage autophagy can be passively activated with the accumulation of dead cells and the disease-related stimulus 7-ketocholesterol (7KC), to exert the function of phagocytosis and clearance [122]. When autophagy is deficient, the activity of NADPH oxidase is enhanced and oxidative stress-inducing factors are upregulated, thereby accelerating apoptosis [120, 123]. Meanwhile, the ability of macrophages to recognize and clear apoptotic cells is impaired, which promotes plaque necrosis [120]. Moreover, macrophage autophagy can also down-regulate the inflammatory response by regulating its own polarization. The most direct evidence is that ATG5 gene knockout mice showed more severe systemic inflammation under a high-fat diet and LPS treatment, and macrophage M1/M2 polarizability was markedly upregulated [124]. However, the activation of autophagy can promote the polarization of macrophages to type M2 and weaken the activation of inflammasome NLRP3, thus stabilizing atherosclerotic plaques [122]. The mechanism by which autophagy inhibits inflammasome may involve autophagy to remove damaged mitochondria (also known as mitophagy), thereby reducing the production of ROS [125]. In addition, lysosome dysfunction is also associated with inflammasome activation [98]. Macrophage autophagy contributes to the maintenance of cholesterol homeostasis. Autophagy facilitates lipid catabolism via lysosomal acid lipase, regulating cholesterol efflux from macrophage foam cells [126]. When lysosomal dysfunction occurs, not only the accumulation of lipids in plaques is increased, but also the proinflammatory nature of cholesterol crystals is further accentuated [98, 127].

VSMCs also play a key role in the development of AS. The increase of VSMC death in the fibrous cap of atherosclerotic lesions causes a destabilization of the plaque. At this point, effective autophagy is particularly important. Autophagy is a key determinant of stress response and phenotypic transition in VSMC, which is activated by various stimuli described above.

Studies have shown that effective autophagy can remove aldehyde-modified proteins to promote VSMC survival [128]. However, the deletion of VSMC-specific autophagy gene ATG7 in mice revealed accelerated cell senescence and death, increased neointima formation following vascular injury, and enhanced AS [129]. More importantly, autophagy defect in VSMCs leads to an imbalance between Ca^2+^ release and reuptake, resulting in an increase in basal Ca^2+^ concentration, thereby enhancing vascular contractility [130]. These data confirm the protective role of VSMC autophagy on atherosclerotic progression. Strategies that stimulate autophagy or reduce the decline in autophagic flux may be beneficial for AS. Studies have found that sirolimus and everolimus can inhibit the autophagy-suppressing kinase mTOR to reduce AS in mice [131, 132].

### Autophagy in VC

Autophagy-lysosomal function plays an important role in regulating VC. The main factor in VC is the release of MVs by VSMCs. These vesicles rich in amorphous Ca^2+^ and Pi, along with enzymes including Phospho-1, tissue non-specific alkaline phosphatase (TNAP), Na^+^/K^+^ ATPase, nucleotide pyrophosphatase phosphodiesterase 1 (NPP1), and PiT-1 are delivered to ECM, where they contribute to the formation of hydroxyapatite [133]. Interestingly, it was found that the MV membrane is rich in phosphatidylethanolamine, which is also a crucial constituent of the autophagosome membrane, and autophagic proteins are expressed on MVs [133]. This suggests that autophagic vesicles can form MVs and release them into the ECM to participate in the occurrence of VC. More importantly, another study uncovered that calcium-containing MVs in osteoblasts were transported via lysosomes and secreted by exocytosis, suggesting that lysosomes play a pivotal role in mineralization [134]. Lysosomal dysfunction causes elevated autophagosome accumulation and increased secretion of MVs, which can aggravate VC [135].

Subsequent studies also identified that the calcification precursors (e.g., calcium and phosphate) are processed in the endosome, autophagosomes, or autolysosomes, participating in the regulation of intracellular calcium and phosphate homeostasis [116]. Furthermore, calcium phosphate precipitation-induced autophagy is dependent on endocytosis. LC3-positive vesicles induced by calcium phosphate precipitates colocalize with ubiquitin and p62, and then colocalize with the lysosomal marker LAMP1, ultimately leading to the completion of the autophagy cycle [136]. Autophagy dysfunction contributes to VC. The fundamental component of VC is hydroxyapatite [137]. The study demonstrated that nano-hydroxyapatite can be internalized into VSMCs to accelerate VC by damaging lysosome function and blocking autophagy flux. The mechanism behind this is mainly that nano-hydroxyapatite inhibits lysosomal acidification, thereby impairing autophagic degradation and promoting the conversion of accumulated autophagic vacuoles into more calcium-containing exosomes released into the ECM [138]. These secreted vesicles tend to aggregate and form microcalcifications, acting as the nucleation sites (Fig. 4) [139].

VSMCs are the critical cell types involved in VC. In addition, our research group demonstrates that calcifying VSMCs are accompanied by mitochondrial dysfunction and decreased levels of autophagy, and the addition of metformin ameliorates VC by inducing mitochondrial biogenesis through mitophagy [140]. Likewise, our other study also reveals that lactate can accelerate VC through BCL2 and adenovirus E1B 19-kDa-interacting protein 3 (BNIP3)-mediated mitophagy deficiency [141]. Beyond that, some studies even have proposed in calcifying skeletal cells that mitochondria can release mitochondrial-derived vesicles (MDVs) rich in Ca^2+^ and Pi, and then these vesicles are engulfed by autophagosomes, or directly taken up by lysosomes where they can be either degraded or released outside the cell [142, 143]. This shows that calcification precursors can also be transferred from dysfunctional mitochondria to lysosomes or ECM, and that mitophagy is likewise involved in the biomineralization process. However, it is not clear in calcifying VSMCs whether mitochondria can also release the MDVs and play a corresponding role (Fig. 4).

## Ferroptosis

### Ferroptotic signaling

**Fig. 5:**
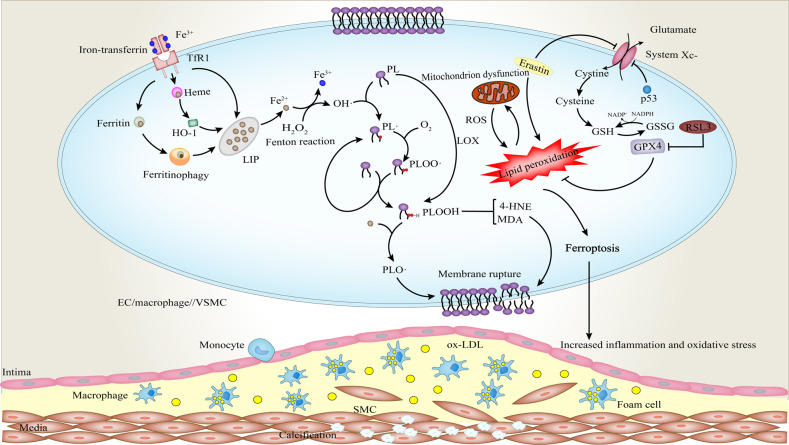
Ferroptotic signaling in AS and VC. Iron-dependent phospholipid peroxidation and reduced antioxidant capacity lead to cell death. Fe^2+^ provided by the labile iron pool (LIP) reacts with hydrogen peroxide (H_2_O_2_) to form the hydroxyl radical (OH·) through Fentons reaction. A carbon-centered lipid radical (PL·) is generated as PUFAs donates hydrogen to (OH·). Then, (PL·) reacts with molecular oxygen (O_2_) to yield a phospholipid peroxyl radical (PLOO·) that abstracts hydrogen from adjacent PUFAs to generate a phospholipid hydroperoxide (PLOOH) and a new (PL·), initiating another lipid radical chain reaction. In enzymatic lipid peroxidation, the generation of PLOOH results from the dioxygenation of PUFAs by lipoxygenases (LOX). PLOOH in the presence of Fe^2+^ can be converted to the alkoxyl phospholipid radical (PLO·), triggering the propagation of lipid peroxidation. In addition, PLOOH can decompose to reactive toxic aldehydes such as 4-hydroxy-2-nonenals (4-HNEs) or malondialdehydes (MDAs) that induce cellular damage. Reduced antioxidant capacity is another important cause of ferroptosis. The inhibition of system Xc^−^ results in the GSH depletion and resultant inactivation of GPX4. Increased lipid peroxidation causes mitochondrial dysfunction, which in turn enhances ROS production and aggravates peroxidative damage. Also, PUFAs mediate inflammatory cascades. These adverse environmental factors together contribute to the progression of AS and the occurrence of VC.

Ferroptosis is a highly iron-dependent form of cell death, which is induced by a variety of mechanisms. Among them, labile iron pool (LIP) plays a crucial role (Fig. 5). Transferrin receptor 1 (TfR1) can recognize the iron-transferrin to mediate cellular iron uptake. The majority of iron is stored in ferritin or heme [144]. However, increased ferritinophagy is able to degrade ferritin and lead to elevated iron levels [145]. Moreover, enhanced activity of heme oxygenase-1 (HO-1) also can cause the excessive activation of heme degradation reaction, thus increasing the release of iron [144]. More importantly, ferroportin (Fpn) is the only known iron transport molecule that can transport ferrous iron (Fe^2+^) from cells to plasma; it can be internalized and degraded by hepcidin, giving rise to the retention of cellular iron with a consequent reduction in systemic iron levels [146]. These factors all will lead to an increase in LIP level, which provides labile iron for Fenton reaction, thereby sensitizing cells to ferroptosis (Fig. 5) [147]. When the intracellular iron is overloaded, most cells do not have the efficient mechanisms of iron export. Iron is a redox-active metal that participates in the formation of oxygen-dependent free radicals and the propagation of lipid peroxidation [148].

Lipid peroxidation is a crucial event in ferroptosis. Polyunsaturated fatty acids (PUFAs) in the membranes are prone to lipid peroxidation, increasing the vulnerability of cells to ferroptosis [149]. In this process, non-enzymatic lipid peroxidation is a free radical-driven chain reaction (Fig. 5). Firstly, hydroxyl radical (OH·) as the most chemically reactive ROS, is generated by the Fenton reaction between Fe^2+^ and hydrogen peroxide (H_2_O_2_) [150]. Secondly, (OH·) abstracts hydrogen from PUFA to form a carbon-centered phospholipid radical (PL·). Thirdly, the rapid reaction of molecular oxygen (O_2_) with the lipid radical yields a phospholipid peroxyl radical (PLOO·). Lastly, (PLOO·) abstracts hydrogen from the adjacent PUFAs, causing the formation of a phospholipid hydroperoxide (PLOOH) and a new (PL·), which can continue to initiate another lipid radical chain reaction. In this auto-amplifying process catalyzed by iron and oxygen, membrane destruction and cell death are triggered, when the molecular regulators of lipid peroxidation are out of balance [150]. In enzymatic lipid peroxidation, lipoxygenases (LOX) can able to catalyze the dioxygenation of PUFAs and produce PLOOH. On the one hand, in the presence of Fe^2+^, PLOOH can be converted to the alkoxyl phospholipid radical (PLO·), which further induces propagation of lipid peroxidation by targeting another PUFA [149, 150]. On the other hand, PLOOH can decompose to reactive toxic aldehydes such as 4-hydroxy-2-nonenals (4-HNEs) or malondialdehydes (MDAs) that inactivate key pro-survival proteins by crosslinking. Continued massive oxidation and consumption of PUFAs may also change membrane fluidity and structure and increase membrane permeability, eventually leading to plasma membrane rupture (Fig. 5) [149, 150].

Another important part of ferroptosis is the weakened ability of antioxidation (Fig. 5). Glutathione (GSH) depletion and subsequent inactivation of glutathione peroxidases 4 (GPX4) ultimately lead to the accumulation of fatal lipid peroxides and the initiation of ferroptosis in the presence of iron [144]. Reduced GSH is the primary intracellular antioxidant in mammals. Cystine/glutamate antiporter (system Xc^−^) imports extracellular cystine in exchange for intracellular glutamate, which plays a key role in maintaining the intracellular GSH levels [150]. However, erastin, as a classic ferroptosis inducer, can affect the synthesis of GSH by suppressing system Xc- to decrease the antioxidant capacity of cells, thus driving cell death. On the other hand, it binds with voltage-dependent anion channels 2/3 to perturb membrane permeability and mediate nicotinamide adenine dinucleotide phosphate hydrides (NADPH) oxidation, which promotes ROS production and mitochondrial damage and eventually leads to ferroptosis [144, 151]. This suggests that the weakened antioxidant effects and enhanced pro-oxidant actions induced by erastin collectively cause cell death. Moreover, in the case of iron overload, most NADPH is used in the Fenton reaction, which hinders the regeneration of GSH. The decrease in the concentration of the reaction substrate GSH facilitates the generation and accumulation of ROS. The presence of more oxidized glutathione (GSSG) also inhibits the synthesis of GSH [144]. These triggers all contribute to a surge in oxidative stress. Importantly, GPX4 is a special member of the selenium-dependent glutathione peroxidase family, which can protect cells against oxidative damage by catalyzing the reduction reactions of H_2_O_2_ and hydroperoxide to produce water or alcohols [152]. Nevertheless, another ferroptosis inducer RSL3 can bind to GPX4 and cause its inactivation, thus inducing lipid peroxidation and cellular membrane damage (Fig. 5) [153]. The depletion or inactivation of GPX4 is considered a fatal signal for ferroptosis [154].

### Ferroptosis in AS and VC

Ferroptosis has positive effects on inflammation [155]. PUFAs and their metabolic enzymes function as facilitators. Arachidonic acid (AA) is a PUFA present in the phospholipids (PL) of cell membranes. When cells are stimulated, AA is released from PL as free AA; it is a precursor of bioactive proinflammatory mediators, such as prostaglandin (PG), IL, TNF, and leukotriene (LT), which promote inflammatory cascades [149].

Iron overload drives the development of AS and VC through its pro-oxidant and proinflammatory effects. Specifically, iron overload impairs EC-derived nitric oxide production and increases ROS, mediating endothelial injury, and monocyte adhesion and transmigration [156]. In human and animal models, compared with healthy arterial tissue, iron levels in atherosclerotic lesions are significantly higher [157]. Excess iron induces the expression of a large number of inflammatory factors (TNF-α, IL-1β, IL-23, IL-10, IL-6, and transforming growth factor β) [158]. Chronic iron overload accelerates the process of AS in apolipoprotein E knockout (ApoE^−/−^) mice by driving oxidative stress and inflammation [159, 160]. Significant shrinkage of mitochondria, and decreased expression levels of SLC7A11(a component of system Xc-) and GPX4 also were found in mouse aortic endothelial cells treated by ox-LDL (Fig. 5). Whereas, the iron chelator deferoxamine can inhibit the production of lipid ROS and MDA to improve cell viability. Likewise, the proinflammatory response such as the upregulation of ICAM1, VCAM1, E-selectin, and MCP-1 also can be reversed by iron chelation in vitro or vivo [160]. Moreover, the addition of ferroptosis inhibitor ferrostatin-1 (Fer-1) downregulates the expressions of adhesion molecules, and upregulates the nitric oxide synthases (eNOS) to prevent endothelial dysfunction; on the other hand, it is able to inhibit iron accumulation, lipid peroxidation and reverse the expression of SLC7A11 and GPX4, alleviating AS lesions in high-fat diet (HFD)-fed ApoE^−/−^ mice [161].

Excess iron also contributes to increased lipid uptake, foam cell formation, polarization, and glycolysis in macrophages [158, 162]. On the one hand, iron load and lipids can inhibit the level of ceruloplasmin in macrophages, which leads to the formation of foam cells [163]; on the other hand, iron overload promotes the production of ROS and accelerates lipid oxidation in foam cells, contributing to the instability of atherosclerotic plaques [158]. In addition, increased aerobic glycolysis in the AS region is considered to be responsible for inflammation and lesion burden. Iron induces polarization of macrophages into a pro-inflammatory macrophage subtype (M1 macrophages) by enhancing glycolysis [158]. M1 macrophages can increase and maintain inflammatory responses by producing pro-inflammatory cytokines [164]. Hepcidin deficiency can reduce macrophage iron and inflammatory phenotype, preventing AS [158]. Similarly, pharmacological suppression of hepcidin increases cholesterol efflux and reduces ROS production and foam cell formation to ameliorate AS, while the administration of exogenous hepcidin reverses this phenomenon [165].

Iron-dependent lipid peroxidation and inflammation also are found in VSMCs. The accumulation of iron in VSMCs provides a prooxidant microenvironment, which promotes the development of foam cells and the vulnerability of plaques [166]. In addition, iron stimulation of VSMCs generates ROS stress through Fenton reaction and increases IL-24 gene expression level, thereby mediating the phenotypic transformation and calcification of VSMCs (Fig. 5). Our previous study also confirmed this. Pro-inflammatory lipid oxidation products like oxidatively modified and acetylated low-density lipoproteins are inducers of VSMC calcification [167]. Enhanced oxidative stress and ROS accumulation run through the entire process of VC. In our experiment, periostin, an important extracellular matrix protein, inhibits the expression of SLC7A11 by down-regulating p53, which contributes to a decrease in the GSH/GSSG ratio, eventually inducing ferroptosis in VSMC [168]. Nevertheless, metformin treatment can partially reverse these changes by activating nuclear factor E2-related factor 2 (Nrf2) signaling, which enhances the antioxidant capacity of VSMCs and alleviates VC. Mechanistically, the ROS-mediated cell damage from lipid peroxidation plays an essential role in the development of VC [169]. These findings might provide insight into the mechanism of VC.

## Conclusion

VC increases the risk of atherosclerotic plaque rupture and cardiovascular death. VSMCs play an integral role in orchestrating VC of both the intima and media. Accompanying the emergence of novel mechanisms that coordinate multiple cell death pathways, the cell fate decision between life and death seems to be more important and relevant to the occurrence and development of various diseases. Timely cell death and clearance are beneficial to the organism, avoiding the possibility of cell infinite proliferation or canceration. However, under pathological conditions, constant stimulation can create feed-forward loops and elicit excessive cell death that ultimately leads to the onset of disease. In VSMC calcification, although autophagy is more beneficial to cell survival and ameliorating VC to some certain extent, most PCD is accompanied by an increase in inflammatory mediators and oxidative stress, which enhances the pro-calcific microenvironments and causes more damage to the tissue. Future efforts need to focus on how PCD can be effectively regulated to reduce the adverse add-on effects of cell death and delay or prevent the progression of calcification, thus reducing cardiovascular risk.

## Data Availability

All data generated or analyzed during this study are included in this published article.
